# TSPY and Male Fertility

**DOI:** 10.3390/genes1020308

**Published:** 2010-09-21

**Authors:** Csilla Krausz, Claudia Giachini, Gianni Forti

**Affiliations:** 1 Andrology Unit, Department of Clinical Physiopathology, University of Florence, Viale Pieraccini 6, Florence 50139, Italy; E-Mail: giachini.claudia@gmail.com; 2 Endocrin Unit, Department of Clinical Physiopathology, University of Florence, Viale Pieraccini 6, Florence 50139, Italy; E-Mail: g.forti@dfc.unifi.it

**Keywords:** TSPY, genetics, male infertility, Y chromosome, spermatogenesis

## Abstract

Spermatogenesis requires the concerted action of thousands of genes, all contributing to its efficiency to a different extent. The Y chromosome contains several testis-specific genes and among them the AZF region genes on the Yq and the *TSPY1* array on the Yp are the most relevant candidates for spermatogenic function. *TSPY1* was originally described as the putative gene for the gonadoblastoma locus on the Y (GBY) chromosome. Besides its oncogenic properties, expression analyses in the testis and *in vitro* and *in vivo* studies all converge on a physiological involvement of the TSPY1 protein in spermatogenesis as a pro-proliferative factor. The majority of *TSPY1* copies are arranged in 20.4 kb of tandemly repeated units, with different copy numbers among individuals. Our recent study addressing the role of *TSPY1* copy number variation in spermatogenesis reported that *TSPY1* copy number influences spermatogenic efficiency and is positively correlated with sperm count. This finding provides further evidence for a role of TSPY1 in testicular germ cell proliferation and stimulates future research aimed at evaluating the relationship between the copy number** and the protein expression level of the* TSPY1* gene.

Spermatogenesis requires the concerted action of thousands of genes, all contributing to its efficiency to a different extent [1]. Male infertility is considered a complex multigenic disease with heterogeneous phenotypical expression ranging from azoospermia to oligozoospermia. On the basis of the currently available data, we can predict that besides highly penetrant mutations in single genes essential to spermatogenesis, the interaction of multiple factors, both genetic and environmental, also should play an important etiopathogenic role. Moreover, although mutations/polymorphisms in genes acting as fine tuners/modulators of the efficiency of spermatogenesis may not necessarily lead to clinically overt conditions [2,3] they are likely to be still relevant to the determination of individual spermatogenic potential. In this regard, a few genetic variants have been reported as significant modulators of sperm production: i) the (CAG)n of exon 1 of the androgen receptor gene [4]; ii) the (TA)n of the *ESR1* gene [5]; and iii) the gr/gr deletion of the Y chromosome [6,7]. The gr/gr deletion removes half of the gene dosage of the AZFc region and represents the first example in andrology of Copy Number Variation (CNV) with clinical significance. Recently, another CNV involving the *TSPY1* array has been reported as a new genetic factor influencing the efficiency of spermatogenesis [8].

*TSPY1* is known as a putative gene for the gonadoblastoma locus on the Y (GBY) chromosome and is abundantly expressed in early stages of tumorigenesis in gonadoblastoma [9,10,11]. Apart from gonadoblastoma, it could be potentially involved in other human cancers, including intracranial germ cell brain tumors [12], hepatocellular carcinoma [13], prostate cancer [14,15] and melanoma [16]. In the testis, TSPY1 has also been reported to be highly expressed in carcinoma *in situ* (CIS) and seminomas and was found to be co-expressed with several germ cell tumor markers [10,11,17,18]. Its oncogenic function is likely to be related to the presence of a SET/NAP domain which serves diverse functions in transcription, translation, DNA replication, signal transduction and cell cycle regulation (for review see Lau *et al.* [10] and references therein). Over-expression of *TSPY1* accelerates G2/M progression of the cell cycle by enhancing the mitotic cyclin B-CDK1 kinase activity [19,20].

Besides its oncogenic properties, it has been postulated that this gene is involved also in physiological functions of the normal testis [21]. In fact, TSPY1 is primarily expressed in gonocytes/prespermatogonia of embryonic testis [22] and in spermatogonia and spermatocytes at meiotic prophase I in adult testis [23]. Various TSPY1 isoforms can be generated by alternative splicing events of transcripts originating from the same transcriptional units. The expression pattern of the isoforms is variable; the full length transcript is expressed predominantly in spermatogonia and the minor *TSPY1* transcript preferentially expressed in round spermatids [15,24]. The role of the isoforms in spermatogenesis, however, remains to be established.

TSPY1 binds both mitotic and meiotic cyclin Bs and, accordingly, it has been reported to co-localize with cyclin B1 in the cytoplasm and nucleus of spermatogonia and of spermatocytes at various maturation stages [19]. The above data suggest that TSPY1 is important for male germ cell replication and renewal in the testis. Its role in early fetal germ cell development has been recently addressed by Schoner *et al.* [25]. The authors provided evidence that TSPY1 is able to partially rescue spermatogenesis and fertility in transgenic Kit*^W-v^*/Kit*^W-v ^*mice.

Other binding partners of TSPY1 are translational elongation factors such as eEF1A1 and eEF1A2 involved in protein synthesis, nuclear export and transcriptional regulation in spermatogonia and spermatocytes [26]. Finally, TSPY1 is also capable of interacting with core histones and thus participating in chromatin modification and/or organization during spermatogenesis [27]. 

Apart from the Y-linked gene, the human genome contains six *TSPY-like* genes on different chromosomes [28]. All members of the *TSPY1* gene family encode protein products that are highly homologous at their carboxylic portions harboring SET/NAP domains. The X-linked single copy homolog, named *TSPX* (*TSPYL2*), contains an acidic domain in the carboxyl terminus which is absent in the other members of the family [29]. The presence of this domain confers contrasting properties in cell cycle regulation and a different expression pattern for TSPX [30,31]. In fact, TSPX interacts with cyclin B and represses the kinase activities of an active cyclin B1-CDK1 complex, acting like a tumor suppressor and resulting in the modulation of cell cycle progression in G2/M phase [19]. *TSPX* is expressed primarily in somatic testis cells (Sertoli cells) and, to a lesser extent, in spermatogonia within the seminiferous tubules [32]. These contrasting properties suggest that TSPY1 and TSPX may be implicated in different functions during normal spermatogenesis. The autosomal *TSPY-like* genes are single-exon genes and could be derived from retrotransposition events involving TSPY transcripts [28]. In a single familial case of sudden infant death syndrome with testicular dysgenesis, a homozygous nonsense mutation that is predicted to result in a truncation of the SET/NAP domain has been described [33]. The affected individuals presented ambiguous genitalia and dysplastic testes with rudimentary fibrotic cords and a diminished number of Leydig and Sertoli cells suggesting a potential involvement of TSPY-L1 in testis differentiation. The function of the remaining four members of the family (*TSPY-L3, 4, 5, 6*) remains unknown.

## *The structure of* TSPY1

*TSPY1* is a tandemly repeated gene and the majority of the *TSPY1* copies are arranged in 20.4 kb of tandemly repeated units, each containing one copy of *TSPY1* and one of the *CYorf16* pseudogene transcription unit, forming a cluster (*DYZ5*) on proximal Yp [34,35]. 

The copy number of *TSPY1*-containing repeat units has been defined in four different studies and ranges from 11 to 76 among individuals [8,36,37,38]. This variation is likely to be generated by unequal sister chromatid exchange [36,39]. Such a structure is highly unusual in the human genome: only 12 protein-coding genes are present in tandem clusters with >3 copies and none of the others shows >16 copies in the current assembly [40]. Maintenance of a minimum *TSPY1* copy number through selection is suggested by the evolutionary conservation of multiple copies of the gene on the Y chromosomes of other mammals [41,42] and by limited variation in copy number in humans, with pronounced modes and few excursions to low or high numbers of copies [43]. The nature of the putative selective force remains entirely unknown, but seems most likely to be related to spermatogenesis.

## *TSPY1* copy number variation and spermatogenesis

The conclusions of these studies are contradictory, mainly due to study design biases (Table 1). In the first study [44], where a higher number of *TSPY1* was found in association with impaired sperm production, the quantification method employed was unable to estimate the exact *TSPY1* copy number since it was not validated against a gold standard method (e.g. pulsed-field gel electrophoresis, PFGE). Moreover, the size of the study population was small (84 cases and 40 controls). The study, which did not find any association between *TSPY1* copy number and infertility, was based on 200 well selected individuals in clinical terms (idiopathic infertile *versus* normozoospermic men), however the authors failed to match cases and controls for Y haplogroup (hg) distribution [38]. The importance of Y haplogroup matching is related to the fact that the mean *TSPY1* copy number differs significantly between Y lineages [8,37] and thus the analysis of *TSPY1* copy number variation in the context of case-control association studies is susceptible to population stratification bias [43]. In fact, Y chromosome distribution varies between different ethnic and geographic areas and a study dealing with unmatched cases and controls has the potential to lead to false conclusions depending on the proportion of Y chromosomes with high and low mean *TSPY1* copy number in each group. Therefore, cases and controls should only differ in their semen phenotype and not in their geographic or ethnic origin, *i.e.,* in their Y chromosome background. 

**Table 1:** Summary of case-control studies dealing with the role of *TSPY1* copy number in male infertility.

**Reference**	**Size of the study population (number of cases-controls)**	Ability to define absolute *TSPY1* copy number*	**Y haplogroup matching between cases and controls**	**Main conclusion**
Vodicka *et al.* 2007 **[44]**	124 (84-40)	no	14 controls *versus* 11 patients	High *TSPY1* copy number is associated with spermatogenic impairment
Giachini *et al.* 2009 **[8]**	284 (154-130)	yes	All controls *versus* all patients	Low *TSPY1* copy number is associated with lower sperm production
Nickkholgh *et al.* 2009 **[38]**	200 (100-100)	yes	Not performed	No association between *TSPY1* copy number and spermatogenic efficiency

* *i.e.*, subjecting the method used for the estimation of the *TSPY1* copy number to prior validation against the gold standard method (pulsed-field gel electrophoresis).

**Figure 1:**
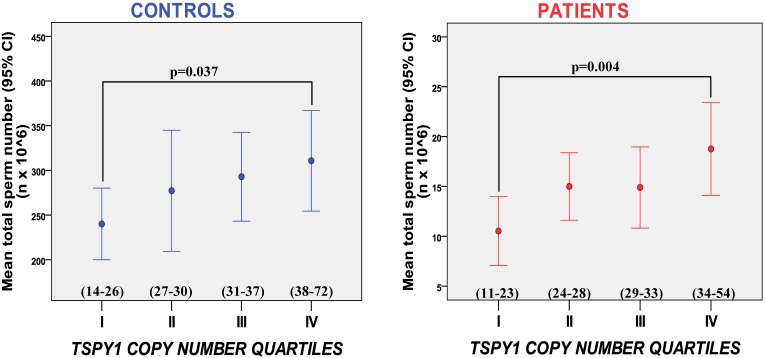
Mean total sperm number in each quartile defined on the basis of* TSPY1* copy number, within patients and controls. The mean total sperm number is significantly higher in the fourth quartile (*i.e.*, in men with higher TSPY copy number) with respect to the first quartile in both groups in our enlarged study population (n = 380).

The only available study free from all the above mentioned selection biases (inclusion of highly selected idiopathic infertile men, normozoospermic controls and Y haplogroup matching of cases and controls) in which a validated analysis against the gold standard method was used, reports a significant association between *TSPY1* copy number and spermatogenic efficiency [8]. In fact, in this study infertile men with abnormal sperm parameters showed a significantly lower number of *TSPY1* copies in respect to men with normozoospermia. The risk of having abnormal sperm parameters was increased 1.5-fold for a man with less than 33 *TSPY1* copies (95% CI 1.2-1.8). As stated above, in order to avoid population stratification, Y haplogroup distribution was strictly matched between cases and controls, therefore the observed association between *TSPY1* copy number and sperm production was genuine, *i.e.,* independent from a Y haplogoup effect. Moreover, this trend toward lower *TSPY1* copy number in the infertile group was observed within each Y haplogroup, suggesting that the contraction or expansion responsible for the observed differences between cases and controls occurs in all Y lineages. Another piece of evidence for a significant contribution of *TSPY1* gene dosage to spermatogenesis is the clear positive correlation observed between sperm count and *TSPY1* copy number both in the infertile and normozoospermic group. Given that the reliability of case-control association studies are strongly dependent on the size of the study population, we are currently increasing the number of subjects in order to verify if our pilot study [8] can be replicated in a larger study population (Supplementary file). The updated results on 380 subjects (212 patients and 168 controls) reinforce our previously published data and show that *TSPY1* copy number does influence sperm production (Figure 1).

## Conclusions

Expression analyses in the testis and *in vitro* and *in vivo* studies all converge on the involvement of the TSPY1 protein in spermatogenesis as a pro-proliferative factor [10,20,25]. On the contrary, the three studies aiming to define the role of *TSPY1* copy number in spermatogenesis reached three different conclusions. This discrepancy is likely to be due to population stratification which representa a crucial bias in case-control association studies aiming to define Y-linked susceptibility factors to impaired sperm production (such as gr/gr deletion and *TSPY1* copy number variation). The only paper free of population stratification bias reported an association between low *TSPY1* copy number and impaired sperm production. The maintenance of the evolutionarily-unstable *TSPY1* array across mammalian species suggests that low copy number must be disadvantageous and this is in line with the finding of a negative effect of low *TSPY1* copy number on spermatogenesis.

We hypothesize that the observed linear correlation between *TSPY1* copy number and sperm count may reflect a high protein expression level in association with high *TSPY1* copy number which would ultimately lead to a higher germ cell proliferation. However, this hypothesis needs to be confirmed by future expression studies aimed to directly correlate TSPY1 expression with *TSPY1* copy number variation. 

## Supplementary Files

Supplementary File 1 PDF-Document (PDF, 18 KB)
