# Supplementary material for: TSPY and Male Fertility
Source: Genes (Basel). 2010 Sep 21;1(2):308–16. doi: 10.3390/genes1020308 (PMC3954084; doi:10.3390/genes1020308)
Supplement: Supplementary File 1 — PDF-Document (PDF, 18 KB) [file genes-01-00308-s001.pdf]

Supplementary

TSPY and Male Fertility

Csilla Krausz, Claudia Giachini and Gianni Forti

Materials and Methods

Subjects

The study population consisted of a total of 380 subjects: 212 idiopathic infertile patients and 168 normozoospermic men (284 subjects: 154 idiopathic infertile patients, and 130 normozoospermic men were already included in our previously published study) [1]. The study was approved by the local ethic committee. Infertile patients included in the study were seeking complete andrological diagnostic work-up for couple infertility at the Andrology Unit and the Unit of Physiopathology of Reproduction of the University Hospital Careggi (Florence). Infertile patients were selected on the basis of a comprehensive andrological examination including medical history, semen analysis, scrotal ultrasound, hormone analysis, karyotype and Y chromosome microdeletion screening. Patients with mono or bilateral cryptorchidism, varicocele grades 2 and 3, obstructive azoospermia, recurrent infections, iatrogenic infertility, hypogonadotrophic hypogonadism, karyotype anomalies, Y chromosome microdeletions including partial deletions of the *AZFc* region, partial AZFc duplications and patients with non-Italian origin were excluded.

According to the three major sperm parameters, the infertile group could be divided as follows: azoospermia in 53 patients; cryptozoospermia (<1 million spermatozoa/mL) in 14 patients; severe oligozoospermia (1–5 million spermatozoa/mL) in 76 patients; moderate oligozoospermia (5–20 million spermatozoa/mL) in 54 patients, astheno and/or teratozoospermia in 15 patients. The mean sperm concentrations in patients and controls were $3.8 \pm 4.4 \times 10^6$ and 86.6 ± 52.4 x 10^6 sperm/mL, respectively. The mean total sperm number in patients and controls were $13.3 \pm 18.3 \times 10^6$ and $278.0 \pm 171.5 \times 10^6$ spermatozoa, respectively. Controls were selected on the basis of normal sperm parameters (sperm count, motility and morphology) defined according to the WHO criteria [2]. Samples were collected using approved protocols and the informed consent of all individuals was obtained.

Estimation of TSPY1 copy number

Relative *TSPY1* copy number was determined by quantitative PCR, using a region of the single copy *PMP22* gene as a control locus as described [1,3]. Absolute numbers of *TSPY1* genes were estimated by reference to DNA samples in which copy number was known from size measurement of the hybridising *XbaI* fragment in pulsed-field gel analysis [4,5].

Detection of the AMELY gene deletion

All patients and controls were screened for *AMELY* gene deletion by PCR amplification of specific STSs: sY70 (GenBank Accession number: G66517) and sY276 (GenBank Accession number: G38362). The amplified products were run on an agarose gel, and all showed the presence of both fragments indicating that *AMELY* was present.

Y haplogroup definition

To exclude recruitment bias, care was taken in the ethnic and geographic matching of the patients and controls. All patients and controls were asked for their paternal and maternal origin and included only if they had Central Italian ancestry on both sides. The subjects were genotyped for six binary markers defining eight haplogroups (including paragroups): hgs A, DE, J, K*(xN, P), N, P*(xR1a), R1a and the remaining Y*(×A, D, E, J, K) chromosomes. Y chromosome haplotyping was performed as previously described for the YAP, M9, *SRY*-1532, 92R7, LLY22g and 12f2 polymorphisms [6]. Polymorphisms were visualized by size or presence/absence of fragments (YAP, 12f2) or restriction enzyme digestion pattern for: M9 (*HinfI*), *SRY*-1532 (*DraIII*), 92R7 and LLY22g (*HindIII*). Further analysis showed that most P*(xR1a) chromosomes fell into R1b1b (M269-derived), but the P*(xR1a) classification is used here because M269 data are not available for all samples.

Statistical analysis

Statistical analysis was performed using the statistical package SPSS for Windows (version 17.0, Chicago, IL, USA). Median and mean values between groups were compared using a nonparametric Mann–Whitney *U* test and Student's *t* test (in case of normal distributions), respectively. Correlation between *TSPY1* copy number and sperm count was ascertained by Spearman's correlation test. The cut off value of *TSPY1* copy number for the comparison of different subgroups was defined on the basis of ROC curve analysis. Differences in the incidence of subjects with *TSPY1* copy number above and below the selected cut off between patients and controls were evaluated using the Fisher's exact test. A *P* value of 0.05 was considered statistically significant for each test.

References and Notes

1. Giachini, C.; Nuti, F.; Turner, D.J.; Laface, I.; Xue, Y.; Daguin, F.; Forti, G.; Tyler-Smith, C.; Krausz, C. TSPY1 copy number variation influences spermatogenesis and shows differences among Y lineages. *J. Clin. Endocrinol. Metab.* **2009**, *94*, 4016-4022.
2. World Health Organization *WHO laboratory manual for the examination and processing of human semen*, 5th ed.; WHO press, Geneva, Switzerland, 2010; pp 223-226.
3. Jobling, M.A.; Lo, I.C.; Turner, D.J.; Bowden, G.R.; Lee, A.C.; Xue, Y.; Carvalho-Silva, D.; Hurles, M.E.; Adams, S.M.; Chang, Y.M.; Kraaijenbrink, T.; Henke, J.; Guanti, G.; McKeown, B.; van Oorschot, R.A.; Mitchell, R.J.; de Knijff, P.; Tyler-Smith, C.; Parkin, E.J. Structural variation on the short arm of the human Y chromosome: recurrent multigene deletions encompassing Amelogenin Y. *Hum. Mol. Genet.* **2007**, *16*, 307-316.

4. Mathias, N.; Bayes, M.; Tyler-Smith, C. Highly informative compound haplotypes for the human Y chromosome. *Hum. Mol. Genet.* **1994**, *3*, 115-123.
5. Oakey, R.; Tyler-Smith, C. Y chromosome DNA haplotyping suggests that most European and Asian men are descended from one of two males. *Genomics* **1990**, *7*, 325-330.
6. Rosser, Z.H.; Zerjal, T.; Hurles, M.E.; Adojaan, M.; Alavantic, D.; Amorim, A.; Amos, W.; Armenteros, M.; Arroyo, E.; Barbujani, G.; *et al.* Y-chromosomal diversity in Europe is clinal and influenced primarily by geography, rather than by language. *Am. J. Hum. Genet.* **2000**, *67*, 1526-1543.

© 2010 by the authors; licensee MDPI, Basel, Switzerland. This article is an open access article distributed under the terms and conditions of the Creative Commons Attribution license (http://creativecommons.org/licenses/by/3.0/).
